# Racial/Ethnic Disparities in Patient Care Experiences among Prostate Cancer Survivors: A SEER-CAHPS Study

**DOI:** 10.3390/curroncol29110659

**Published:** 2022-11-01

**Authors:** Ambrish A. Pandit, Laura E. Gressler, Michael T. Halpern, Mohamed Kamel, Nalin Payakachat, Chenghui Li

**Affiliations:** 1 Department of Pharmacy Practice, University of Arkansas for Medical Sciences, Little Rock, AR 72211, USA; 2 Division of Cancer Control and Population Sciences, National Cancer Institute, Bethesda, MD 20892, USA; 3 College of Medicine, University of Cincinnati, Cincinnati, OH 45267, USA; 4 Department of Urology, Ain Shams University, Cairo 11566, Egypt

**Keywords:** patient care experiences, prostate cancer, disparity, SEER, CAHPS

## Abstract

Purpose: To evaluate racial/ethnic disparities in patient care experiences (PCEs) among prostate cancer (PCa) survivors. Methods: This retrospective study used 2007–2015 National Cancer Institute Surveillance, Epidemiology and End Results registry data linked to Consumer Assessment of Healthcare Providers and Systems surveys. First survey ≥ 6 months post-PCa diagnosis was analyzed. We performed multivariable linear regression, adjusting for demographic and clinical covariates, to evaluate the association of race/ethnicity (non-Hispanic Whites (NHWs), non-Hispanic Black (NHBs), Hispanic, non-Hispanic Asian (NHAs), and other races) with PCE composite measures: getting needed care, doctor communication, getting care quickly, getting needed prescription drugs (Rx), and customer service. Results: Among 7319 PCa survivors, compared to NHWs, Hispanics, NHBs and NHAs reported lower scores for getting care quickly (ß = −3.69; *p* = 0.002, ß = −2.44; *p* = 0.021, and ß = −6.44; *p* < 0.001, respectively); Hispanics scored worse on getting needed care (ß = −2.16; *p* = 0.042) and getting needed Rx (ß = −2.93; *p* = 0.009), and NHAs scored worse on customer service (ß = −7.60; *p* = 0.003), and getting needed Rx (ß = −3.08; *p* = 0.020). However, NHBs scored better than NHWs on doctor communication (ß = 1.95, *p* = 0.006). No statistically significant differences were found between other races and NHWs. Conclusions: Comparing to NHWs, Hispanics and NHAs reported worse experiences on several PCE composite measures, while NHBs reported worse scores on one but better scores on another PCE composite measure. Further research is needed to understand the reasons behind these disparities and their influence on healthcare utilization and health outcomes among PCa survivors.

## 1. Introduction

The National Academy of Medicine, formerly known as the Institute of Medicine, defines ‘disparities’ as differences in quality of healthcare that are not attributable to access-related factors, clinical needs, or patient preferences [1]. Establishing health equity is one of the overarching goals of the Healthy People 2030 initiative [2]. Cancer health equity is considered a guiding principle by The American Society of Clinical Oncology (ASCO) [3]. Race/ethnicity continues to be a main driver of disparities and poses a significant risk to health equity and quality of care among different population sub-groups [1]. Disparities in activities across the cancer care continuum can lead to differences in cancer outcomes [3].

Quality of care, measured by quality measures [4], is greatly influenced by racial/ethnic disparities [1]. Patient care experiences (PCEs), a quality measure integral to healthcare quality, cover a wide range of patient interactions with healthcare-system stakeholders and components. This includes providers, nurses, healthcare facilities staff, hospital, medical insurance plans, and drug plans [5]. PCEs encompass aspects of care such as access to care, timeliness of care, patient–provider communication, etc., that are highly valued by patients [5]. Racial disparities in PCEs have been previously studied among the general population [6], and cancer survivors [7].

Prostate cancer (PCa) is one of the most prevalent cancers affecting men and also a leading cause of cancer-related deaths among men in the United States [8]. PCa often progresses slowly with a five-year survival rate of >95% [9]. Prostate cancer that is localized to the prostate gland is usually treated with surgery and/or radiation or managed through active surveillance [10]. Patients with PCa in a more advanced stage work with clinicians to manage their disease through hormone therapy or chemotherapy [11]. Whatever the treatment/management modality involved, follow-up care becomes crucial for PCa survivors. PCa-related treatment/management involves frequent interactions with healthcare providers and systems [12,13]. Given their frequent interactions with the healthcare ecosystem, studying racial/ethnic disparities in PCEs among PCa survivors is crucial.

To our best knowledge, only one population-based study has evaluated racial/ethnic disparities in PCEs among U.S. PCa survivors [14]. The study leveraged the Surveillance, Epidemiology, and End Results (SEER) data linked to Consumer Assessment of Healthcare Providers and Systems (CAHPS) database between 2000 and 2011. The study found that compared to NHWs, Hispanics and NHAs reported worse PCEs for getting care quickly, doctor communication, and getting needed prescription drugs but NHBs reported better PCEs for getting needed care, and customer service. A re-evaluation of racial/ethnic disparities in PCEs using more recent data is needed given that prostate cancer care has changed significantly in the past decade in the wake of screening [15,16] and treatment [11,17,18,19] guidelines changes, new technology [20,21,22,23], and care delivery practices [24,25]. Thus, the objective of this study was to evaluate racial/ethnic disparities in PCEs of PCa survivors.

## 2. Materials and Methods

### 2.1. Study Dataset

This study used the SEER-CAHPS data between 2007 and 2015 [26]. SEER is a population-based cancer registry that provides information on patient demographics as well as cancer-related clinical information such as tumor primary site, stage, morphology, first course of treatment, and follow-up for vital status [27]. CAHPS are surveys administered to Medicare enrollees and capture their demographics and healthcare experiences [28]. Medicare enrollment data of PCa survivors were also available as a part of SEER-CAHPS dataset [29]. The United States’ Federal Information Processing Standards (FIPS) codes from CAHPS surveys were also linked to the United States Department of Agriculture Economic Research Service Rural-Urban Continuum Codes (RUCC) [30] to determine rurality of survivors’ residence.

### 2.2. Study Population

CAHPS surveys Medicare enrollees about their care experiences within a period of 6 months preceding the survey. To ensure capturing of PCEs post PCa diagnosis, the study population included PCa survivors having completed at least one CAHPS survey ≥ 6 months after a PCa diagnosis. For survivors with multiple surveys ≥ 6 months after PCa diagnosis, the first survey was analyzed. We excluded individuals: (1) with missing month/year of PCa diagnosis; (2) missing survey date; (3) who were diagnosed at autopsy or through death certificate; (4) with missing race/ethnicity information; and (5) without a valid score for any of the PCE composite measures.

### 2.3. Exposure

#### Race/Ethnicity

The primary exposure was race/ethnicity, classified into five mutually exclusive categories (non-Hispanic Whites (NHW), non-Hispanic Blacks (NHB), Hispanics, non-Hispanic Asians (NHA), and other races) based on information available from CAHPS. For individuals with missing race/ethnicity information in CAHPS, we used the race/ethnicity information from SEER; if that too was missing, we extracted race/ethnicity information from the Medicare enrollment database. Individuals missing race/ethnicity information from the CAHPS, SEER, and Medicare enrollment database were excluded from the study sample. We used NHW as reference category and all other race/ethnicity categories were compared to NHW.

### 2.4. Outcome Variables

The outcomes of interest were five PCE composite measures from CAHPS. These were ‘getting needed care’, ‘getting care quickly’, ‘physician communication’, ‘getting needed prescription drugs’, and ‘customer service’. CAHPS uses a linear mean scoring method [31] to provide the composite measures scores ranging from 0 to 100. Consistent with previous research, differences in PCE scores under 3 points were considered ‘small’, ≥3 but <5 points were considered ‘medium’, while ≥5 points were considered ‘large’ differences [32]. Further details are provided in e-Methods.

### 2.5. Covariates

We adjusted all models for the following survivors’ demographic and clinical characteristics. We included the SEER-CAHPS recommended case-mix variables: age when responded to survey; proxy answering questions for respondent; mental health status; general health status; low-income subsidy; dual eligibility; and education [31]. Additionally, based on variables identified as potential confounders in the prior literature, we adjusted for plan type [14], prescription drug plan [33], marital status [33], geographic region of residence at the time of CAHPS survey [34], urban/rural residence status [34], Census Tract Poverty Indicator for neighborhoods [34], survey year [14], current smoking status [34], tumor grade, lymph node involvement, risk of disease progression, receipt of radiation as a part of initial treatment, receipt of definitive surgery as a part of initial treatment, number of prior cancers other than prostate cancer [14], time between prostate cancer diagnosis and CAHPS survey [14], and comorbidity count [34]. Further details are provided in e-Methods.

### 2.6. Statistical Analysis

We compared the demographics and clinical characteristics of PCa survivors by race/ethnicity categories, using chi-square test and Fisher’s exact test as appropriate for categorical variables and ANOVA test for continuous variables. We conducted multicollinearity testing for the covariates mentioned above (Table S1). A Variance Inflation Factor (VIF) of >10 for any covariate was considered as a sign of multicollinearity. We performed multivariable linear regression modelling to evaluate the association of race/ethnicity with each PCE composite measure, adjusting for covariates mentioned above (fully adjusted models), models adjusted for SEER-CAHPS recommended case-mix variables (partially adjusted models), and unadjusted models. We only performed complete case analyses, hence the sample sizes varied for models of different PCE measures. We used SAS v.9.4 to perform statistical analysis. This research was determined to be non-Human subject research by the University of Arkansas for Medical Sciences Institutional Review Board (IRB # 260675).

## 3. Results

### 3.1. Study Cohort and Demographics

**Figure 1:**
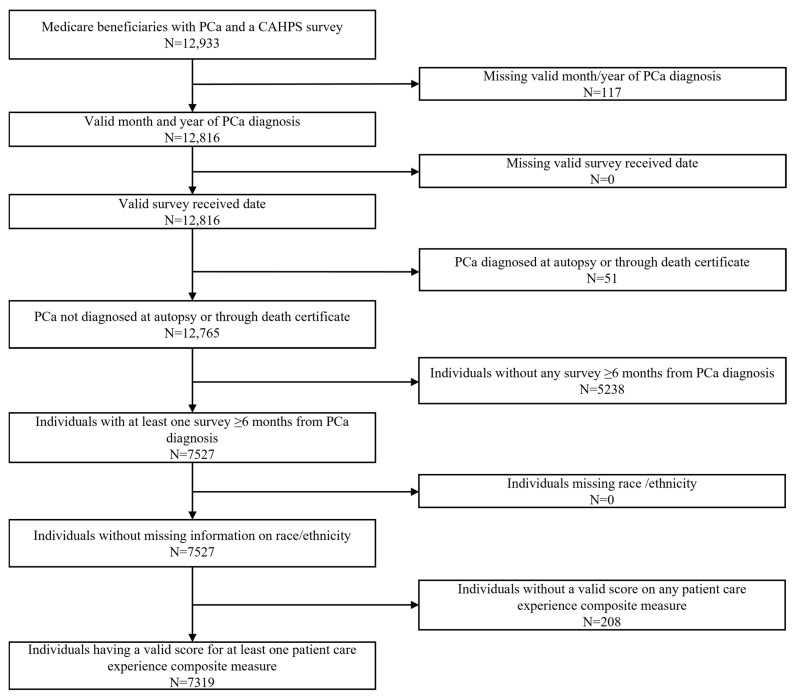
Sample selection flow chart for study sample. PCa: Prostate cancer; CAHPS: Consumer Assessment of Healthcare Providers and Systems.

The study sample included 7319 PCa survivors after applying the inclusion and exclusion criteria. Figure 1 provides details of sample selection. Race/ethnicity information was missing in CAHPS for 420 individuals and was obtained from SEER or Medicare enrollment file. No individuals were excluded due to missing race/ethnicity information after using all three sources.

The sociodemographic characteristics of our study sample are presented in Table 1. In the study sample, 5253 (71.7%) were NHWs, 851 (11.6%) were NHBs, 595 (8.1%) were Hispanics, 386 (5.3%) were NHAs, and 234 (3.2%) belonged to other races. While 52.8% of NHW PCa survivors were enrolled in FFS plans, 65.5%, 72.1%, 63.5%, and 53.0% of NHB, Hispanic, NHA, and other races PCa survivors, respectively, were enrolled in MA plans (*p* < 0.001). A higher proportion of NHW, NHA, and other races PCa survivors (63.5%, 55.4%, and 53.0%, respectively) reported having some college or higher education compared to only 34.9% of NHB and 30.1% of Hispanic PCa survivors, respectively (*p* < 0.001). Compared to 5.6% NHW PCa survivors, 15.8% PCa survivors from other races had low-income subsidy; this proportion was even bigger for NHAs (24.4%), NHBs (29.7%), and highest at 34.8% for Hispanics (*p* < 0.001). No statistically significant differences were found by race categories with regards to time between PCa diagnosis and CAHPS survey.

Clinical characteristics by race/ethnicity categories are reported in Table 2. Despite the fact that the majority of PCa survivors had received either definitive surgery or radiation as part of the initial treatment (>60% for all race/ethnicity categories), no lymph node involvement (>80% for all race/ethnicity categories), and no other cancers (>88% for all race/ethnicity categories), there are significant (*p* < 0.05) differences in these variables by race/ethnicity groups (Table 2). Most PCa survivors (>50% across all race/ethnicity categories) had poorly differentiated tumors. Except for Hispanics, >50% PCa survivors from all other race/ethnicity categories had an intermediate risk of disease progression (*p* < 0.05) (Table 2).

Across all race/ethnicity categories, >60% and >75% PCa survivors reported excellent/very good/good general health status and mental health status, respectively (Table 3).

### 3.2. Regression Analyses

The least-square mean estimates (LSM) from unadjusted, partially adjusted, and fully adjusted multivariable linear regression analysis for each PCE measure by race/ethnicity categories show that for each PCE composite measure, the LSM from unadjusted models was higher than the respective LSM from partially and fully adjusted models (Table 4). Getting care quickly had the lowest average scores among all outcome measures and across all race/ethnicity categories (Table 4).

**Figure 2:**
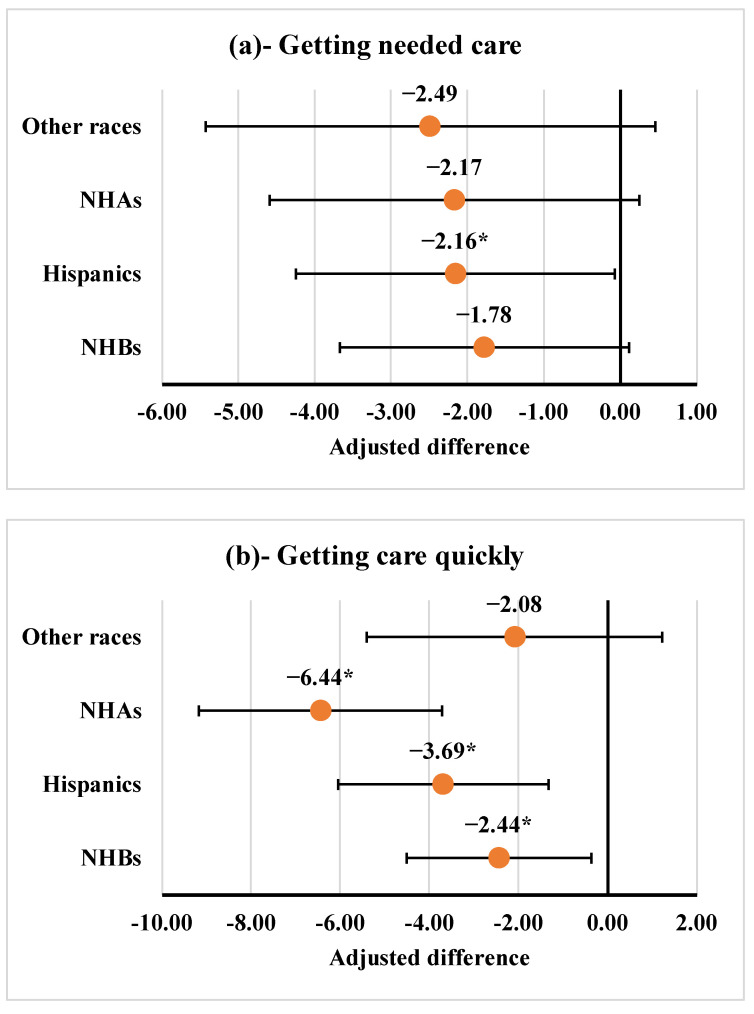

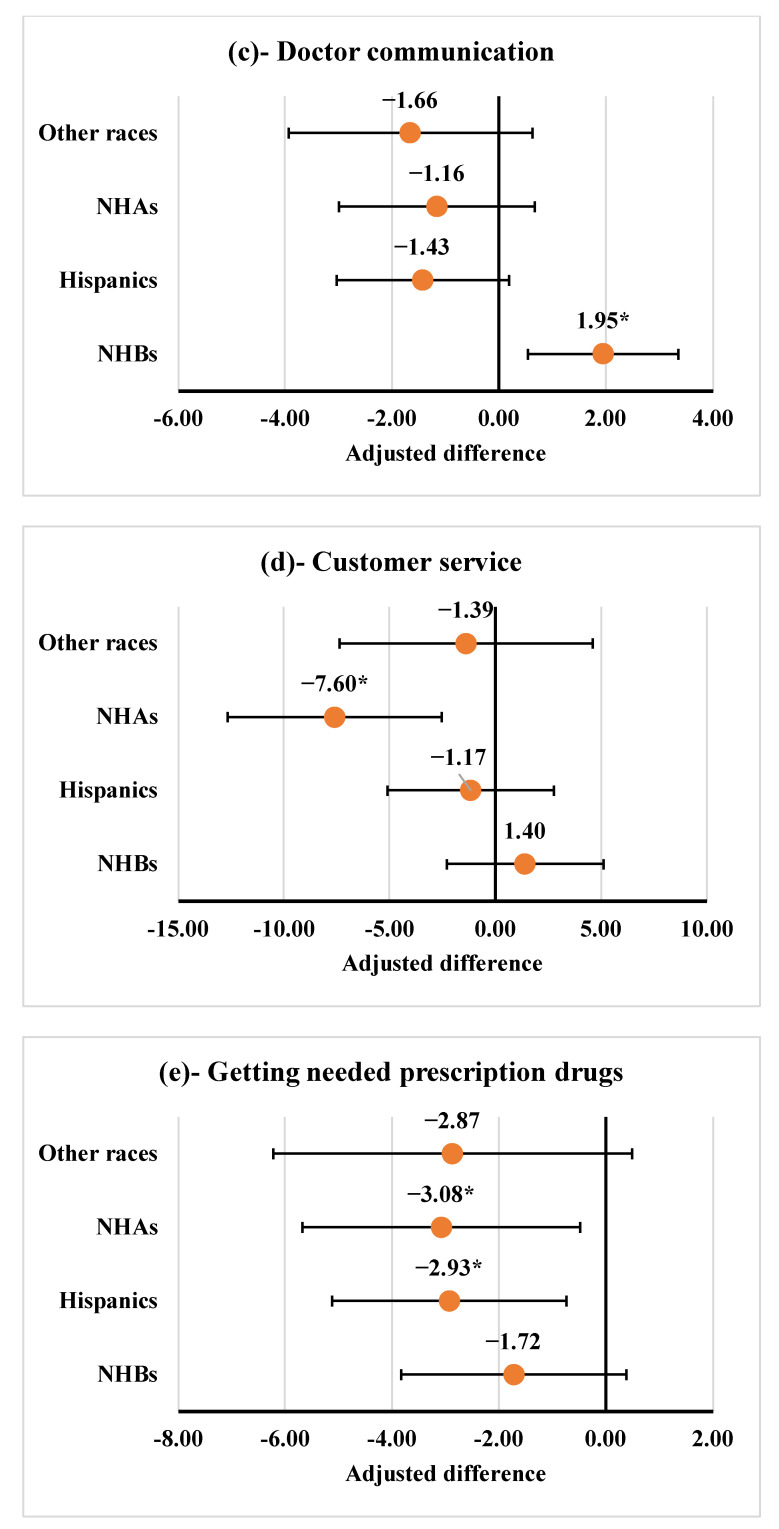
Adjusted differences in least-square mean estimates of patient experience measures by race/ethnicity. * Statistically significant difference at *p* < 0.05 level. NHAs: non-Hispanic Asians; NHBs: non-Hispanic Blacks; N = 5486 for getting needed care, N = 6323 for getting care quickly, N = 5487 for doctor communication, N = 1968 for customer service, and N = 4131 for getting needed prescription drugs. The error bars indicate 95% confidence intervals around the point estimate. Note: Adjusted difference between least-square mean estimate of a race/ethnicity category compared to non-Hispanic Whites derived from fully adjusted models. Models adjusted for case-mix variables (age when responded to survey, proxy answering questions for respondent, mental health status, general health status, low income subsidy, dual eligibility, and education), adjusted for plan type, prescription drug plan, marital status, geographic region of residence at the time of CAHPS survey, urban/rural residence status, Census Tract Poverty Indicator for neighborhoods survey year, current smoking status, tumor grade, lymph node involvement, risk of disease progression, receipt of radiation as a part of initial treatment, receipt of definitive surgery as a part of initial treatment, number of prior cancers other than prostate cancer, time between prostate cancer diagnosis and CAHPS survey, comorbidity count.

Adjusted mean differences and 95% confidence intervals (CIs) from fully adjusted multivariable linear regression analyses for each PCE composite measure by race/ethnicity categories are presented in Figure 2 (panels 2a–2e). Getting care quickly was the only outcome measure where three race/ethnicity categories (NHB, Hispanic, and NHA) reported significantly lower scores as compared to NHW. We found that Hispanic PCa survivors, compared to NHW PCa survivors, reported significantly lower scores for getting needed care (ß = −2.16, 95% CI −4.25 to −0.07; *p* = 0.042) (Figure 2a), getting care quickly (ß = −3.69, 95% CI −6.05 to −1.33; *p* = 0.002) (Figure 2b), and getting needed prescription drugs (ß = −2.93, 95% CI −5.12 to −0.74; *p* = 0.009) (Figure 2e). Similarly, NHA PCa survivors, compared to NHW PCa survivors, reported significantly lower scores for getting care quickly (ß = −6.44, 95% CI −9.17 to −3.70; *p* < 0.001) (Figure 2b), customer service (ß = −7.60, 95% CI −12.67 to −2.53; *p* = 0.003) (Figure 2d), and getting needed prescription drugs (ß = −3.08, 95% CI −5.68 to −0.48; *p* = 0.020) (Figure 2e). Although NHB PCa survivors reported significantly lower scores for getting care quickly (ß = −2.44, 95% CI −4.52 to −0.37; *p* = 0.021) than NHW PCa survivors (Figure 2b), they reported significantly higher score for doctor communication (ß = 1.95, 95% CI 0.55 to 3.36; *p* = 0.006) (Figure 2c). No significant differences in PCE composite measures were found between PCa survivors of other races and NHW survivors.

## 4. Discussion

This study evaluating racial disparities in PCEs of PCa survivors found that race/ethnicity, even after adjusting for a comprehensive list of demographic, socioeconomic, and clinical characteristics, was significantly associated with several PCEs. Out of the five PCE composite measures, Hispanic and NHA PCa survivors compared to NHW PCa survivors reported significantly worse adjusted scores on three measures each, while similar scores for other PCEs. Hispanic compared to NHW PCa survivors reported significantly poorer care experiences for getting needed care, getting care quickly, and getting needed prescription drugs. NHA compared to NHW PCa survivors reported significantly poorer experiences for getting care quickly, customer service, and getting needed prescription drugs. An evaluation of racial disparities between NHB and NHW PCa survivors found mixed results. NHB compared to NHW PCa survivors reported significantly poorer adjusted scores for getting care quickly, but significantly better scores on doctor communication, and similar scores in the other PCE composite measures.

### 4.1. Racial Disparities in PCE Composite Measures

#### 4.1.1. Racial Disparities in Getting Needed Care

Access to healthcare is at the core of quality healthcare and disparities in healthcare access indicate poor quality healthcare [35]. Getting needed medical care, prescription drugs, preventive services, screenings, and timeliness of care are components of access to healthcare [36]. The Centers for Medicare and Medicaid Services (CMS) continuously strive for healthcare equity across different racial/ethnic groups to improve efficiency of healthcare systems and promote access to high quality healthcare through affordable coverage [37]. We observed that Hispanic, compared to NHW PCa survivors, on average reported poorer adjusted scores for getting needed care. This coincides with the observation that 72.1% Hispanic compared to 47.2% NHW PCa survivors were enrolled in any MA plan. Getting needed care has been associated with health plan type [38]. MA compared to FFS enrollees have traditionally reported significantly poor access to care, and lower scores for getting needed care [39,40]. However, we found that after adjusting for race/ethnicity and other covariates, MA plans were not different from FFS plans in PCE measures except customer service and getting needed prescription drugs. MA plans reported a higher adjusted score for getting needed prescription drugs. Despite adjusting for an extensive list of survivors’ sociodemographic and clinical characteristics in our regression models, significantly worse adjusted scores for getting needed care persisted among racial/ethnic minorities compared to NHW survivors. This supports previous findings that racial/ethnic disparities exist independent of disparities in other factors associated with healthcare access [1].

#### 4.1.2. Racial Disparities in Getting Care Quickly

All racial/ethnic minorities, except the other races group, reported significantly lower adjusted scores compared to NHWs for getting care quickly. Moreover, getting care quickly had the lowest adjusted average scores among all PCEs and across all races. According to the CMS Office of Minority Health Report assessing trends in racial inequities in healthcare from 2009–2018 among a nationally representative sample of MA enrollees, NHB, Hispanic, and NHA enrollees consistently reported lower adjusted scores for getting care quickly [41]. The identified patterns of disparities for adjusted scores of getting care quickly per this report are NHA-NHW (maximum negative difference) > Hispanic-NHW > NHB-NHW (minimum negative difference). This is very similar to the patterns observed in our study. Low average scores and observed disparities that span across multiple racial/ethnic minority groups warrant particular attention to disparities in getting care quickly.

#### 4.1.3. Racial Disparities in Doctor Communication

Compared to NHWs, NHB PCa survivors reported higher adjusted scores for doctor communication. There is conflicting evidence regarding doctor communication among NHBs. Some studies reported NHB race to be associated with poorer doctor communication among breast cancer survivors [42] and in the general population [43], while others found NHBs to be associated with better doctor communication among Medicare and Medicaid enrollees [40,44]. These findings suggest that racial disparities in doctor communication may vary by cancer site, gender, or insurance type. Further research is required to fully understand racial/ethnic disparities in doctor communication.

#### 4.1.4. Racial Disparities in Customer Service and Getting Needed Prescription Drugs

NHAs on average reported poorer experiences (7.6 points lower adjusted difference) with health plan customer service when compared to NHWs. This finding is consistent with two CMS reports on trends in racial inequities in healthcare among Medicare enrollees [41,45], which found that NHA enrollees reported 6 to 11 points lower scores than NHW enrollees [41,45]. Furthermore, our findings of lower average adjusted scores for getting needed prescription drugs among Hispanic and NHA compared to NHW PCa survivors are in concordance with previous studies of cancer patients, which reported that Hispanic [14] and NHAs [14,46] (vs. NHWs) were less likely to give excellent/high scores (score of 100) for getting needed prescription drugs, and further substantiated by the CMS reports among general MA population. [6,45] Although previous research has pointed out that NHAs are likely to have poorer care experiences [43,44], other studies suggest that at least some of the NHA-NHW differences may be attributable to less use of extreme response options by NHA compared to NHW [47,48]. More research is needed to identify contributing factors such as cultural influences and medical system factors.

### 4.2. Comparison to Previous Study in PCa Survivors Using SEER-CAHPS

Using 2007–2015 SEER-CAHPS data, this study builds on the existing evidence [14] of racial/ethnic disparities in PCEs among PCa survivors. It is important to note that the previous study dichotomized the PCE outcomes as ‘high/not high’, while our study analyzed PCE scores/ratings as continuous measures, which has greater statistical power [31]. Moreover, survey questions for many of the PCEs have evolved over the years, from 2000 to 2015 [28], making it impossible to directly compare with previous study findings. Nonetheless, the direction of many of the adjusted associations observed in our study were consistent with those previously reported [14]. For instance, in the same way as the previous study [14], we found that compared to NHW PCa survivors, Hispanic and NHA PCa survivors had poorer experiences with getting care quickly and getting needed prescription drugs. However, there were some inconsistencies as well, especially regarding PCEs in NHBs. Further details are provided in Table S2 and e-Methods.

### 4.3. Limitations

This study has several limitations that should be considered when interpreting the findings. First, the study sample included PCa survivors residing in SEER regions who completed a Medicare CAHPS survey ≥ 6 months after diagnosis and estimates and were not weighted. In addition, the Medicare CAHPS process over-samples MA enrollees relative to FFS [49]. Therefore, the study results may not be generalized to all PCa survivors or all Medicare enrollees, or PCa survivors in other countries. CAHPS surveys reflect on the care received within 6 months prior to completing the survey and may represent PCEs in different cross-sections of survivorship depending on when the survey was taken. Since PCa survivors’ healthcare needs and use of health services, and correspondingly care experiences may change over time, the reported PCEs may pertain to elements of care that are not PCa-specific treatment. We used self-reported race/ethnicity information from CAHPS as the primary source. A study has shown that individuals of racial/ethnic minorities have a higher tendency to skip race/ethnicity questions in surveys [50], which may bias the distribution of race/ethnicity, leading to an underrepresentation of minority survivors. However, after supplementing this with information used from the SEER and/or Medicare enrollment file, there were no individuals excluded due to missing race.

## 5. Conclusions

This study provides important insights about racial/ethnic disparities in PCEs among PCa survivors. Specifically, both Hispanic and NHA PCa survivors on average had significantly poorer care experiences than NHW PCa survivors for multiple PCE measures. Results on NHBs were mixed. Disparities in getting care quickly were recorded for all racial/ethnic minorities except the other races group. While studies on racial disparities in care experiences focused mostly on improving care experiences among racial/ethnic minorities, the aspects of PCEs where NHWs perform poorer than racial/ethnic minorities, such as doctor communication, should also be further investigated. Additionally, a more detailed assessment of racial/ethnic disparities where multiple race/ethnicity groups report low scores such as getting care quickly and getting needed prescription drugs should be conducted. Consistent with the previous definition of racial/ethnic disparities [1], we found that race/ethnicity was associated with disparities in reported PCEs even after adjusting for a comprehensive list of survivors’ sociodemographic, clinical, and health-related factors. Further research is urgently needed to identify the sources of these residual disparities which could be attributed to provider-, institution-, and health system-level factors that we did not study. Measures targeting these sources may be formulated to minimize the negative impact of such disparities [51]. In addition, research is required to evaluate how racial/ethnic disparities in PCEs influence healthcare utilization and health outcomes among PCa survivors.

## Figures and Tables

**Table 1:** Sociodemographic characteristics of study cohort by race/ethnicity ^a^.

Variable	Non-Hispanic Whites (*n* = 5253)	Non-Hispanic Blacks (*n* = 851)	Hispanics (*n* = 595)	Non-Hispanic Asians (*n* = 386)	Other Races (*n* = 234)	*p*-Value
**Age (in years) when responded to survey, Mean ± SD ^b^**	75.11 ± 6.54	72.48 ± 6.83	73.85 ± 6.70	76.17 ± 6.42	73.47 ± 6.60	<0.001
**Education level, *n* (%)**						<0.001
Some college/higher	3335 (63.5)	297 (34.9)	179 (30.1)	214 (55.4)	124 (53.0)	
High school/less	1670 (31.8)	500 (58.8)	377 (63.4)	146 (37.8)	>99 (>42.3)	
Missing	248 (4.7)	54 (6.4)	39 (6.6)	26 (6.7)	<11 (<4.7)	
**Marital status, *n* (%)**						<0.001
Not married	844 (16.1)	256 (30.1)	120 (20.2)	46 (11.9)	52 (22.2)	
Married	3628 (69.1)	460 (54.1)	347 (58.3)	283 (73.3)	139 (59.4)	
Missing	781 (14.9)	135 (15.9)	128 (21.5)	57 (14.8)	43 (18.4)	
**Health plan type at the time of CAHPS ^c^ survey, *n* (%)**						<0.001
Fee-for-service	2773 (52.8)	294 (34.6)	166 (27.9)	141 (36.5)	110 (47.0)	
Medicare Advantage	2480 (47.2)	557 (65.5)	429 (72.1)	245 (63.5)	124 (53.0)	
**Prescription drug plan, *n* (%)**						<0.001
No	2262 (43.1)	264 (31.0)	121 (20.3)	102 (26.4)	87 (37.2)	
Yes	2991 (56.9)	587 (69.0)	474 (79.7)	284 (73.6)	147 (62.8)	
**Low-income subsidy, *n* (%)**						<0.001
No	4960 (94.4)	598 (70.3)	388 (65.2)	292 (75.7)	197 (84.2)	
Yes	293 (5.6)	253 (29.7)	207 (34.8)	94 (24.4)	37 (15.8)	
**Dual eligibility for Medicare and Medicaid, *n* (%)**						<0.001
No	4984 (94.9)	649 (76.3)	419 (70.4)	298 (77.2)	199 (85.0)	
Yes	226 (4.3)	>191 (>22.4)	>165 (>27.7)	>77 (>19.9)	>24 (>10.3)	
Missing	43 (0.8)	<11 (<1.3)	<11 (<1.8)	<11 (<2.8)	>11 (<4.7)	
**Census tract poverty indicator, *n* (%)**						<0.001
Neighborhoods with 0%–<5% poverty	1618 (30.8)	76 (8.9)	72 (12.1)	126 (32.6)	51 (21.8)	
Neighborhoods with 5% to <10% poverty	1569 (29.9)	145 (17.0)	116 (19.5)	130 (33.7)	62 (26.5)	
Neighborhoods with 10% to <20% poverty	1455 (27.7)	232 (27.3)	205 (34.5)	89 (23.1)	74 (31.6)	
Neighborhoods with 20% to 100% poverty	571 (10.9)	>387 (>45.5)	>191 (>32.1)	>30 (>7.8)	>36 (>15.4)	
Missing	40 (0.8)	<11 (<1.3)	<11 (<1.8)	<11 (<2.8)	<11 (4.8)	
**Proxy answered questions for the respondent, *n* (%)**						<0.001
No	4283 (81.5)	553 (65.0)	360 (60.5)	282 (73.1)	166 (70.9)	
Yes	365 (7.0)	121 (14.2)	143 (24.0)	60 (15.5)	22 (9.4)	
Missing	605 (11.5)	177 (20.8)	92 (15.5)	44 (11.4)	46 (19.7)	
**Current smoking status, *n* (%)**						<0.001
Non-smoker	4567 (86.9)	660 (77.6)	519 (87.2)	343 (88.9)	197 (84.2)	
Smoker	467 (8.9)	139 (16.3)	50 (8.4)	17 (4.4)	>26 (>11.1)	
Missing	219 (4.2)	52 (6.1)	26 (4.4)	26 (6.7)	<11 (<4.7)	
**Geographic region when answering to CAHPS ^c^ survey, *n* (%)**						<0.001
Northeast	904 (17.2)	138 (16.2)	>89 (>14.6)	15 (3.9)	21 (9.0)	
Midwest	619 (11.8)	92 (10.8)	<11 (<1.8)	<11 (<2.8)	15 (6.4)	
South	1091 (20.8)	449 (52.8)	42 (7.1)	12 (3.1)	41 (17.5)	
West	2639 (50.2)	>161 (>18.9)	>442 (>74.3)	>348 (>90.2)	157 (67.1)	
Missing	0 (0.0)	<11 (<1.3)	<11 (<1.8)	0 (0.0)	0 (0.0)	
**Rurality, *n* (%)**						<0.001
Urban	4575 (87.1)	781 (91.8)	553 (92.9)	371 (96.1)	198 (84.6)	
Rural	>667 (>12.7)	>59 (>6.9)	42 (7.1)	15 (3.9)	36 (15.4)	
Missing	<11 (<0.2)	<11 (<1.3)	0 (0.0)	0 (0.0)	0 (0.0)	
**Survey year, *n* (%)**						0.053
2008	186 (3.5)	29 (3.4)	24 (4.0)	17 (4.4)	<11 (<4.7)	
2009	390 (7.4)	78 (9.2)	51 (8.6)	29 (7.5)	>27 (>11.5)	
2010	532 (10.1)	96 (11.3)	64 (10.8)	57 (14.8)	28 (12.0)	
2011	700 (13.3)	106 (12.5)	56 (9.4)	40 (10.4)	25 (10.7)	
2012	872 (16.6)	132 (15.5)	102 (17.1)	66 (17.1)	43 (18.4)	
2013	928 (17.7)	133 (15.6)	105 (17.7)	73 (18.9)	36 (15.4)	
2014	883 (16.8)	162 (19.0)	114 (19.2)	62 (16.1)	36 (15.4)	
2015	762 (14.5)	115 (13.5)	79 (13.3)	42 (10.9)	28 (12.0)	
**Time between PCa ^d^ diagnosis and CAHPS ^c^ survey, *n* (%)**						0.320
Less than 2 years	1940 (36.9)	322 (37.8)	240 (40.3)	150 (38.9)	100 (42.7)	
2–5 years	2418 (46.0)	380 (44.7)	255 (42.9)	171 (44.3)	88 (37.6)	
>5 years	895 (17.0)	149 (17.5)	100 (16.8)	65 (16.8)	46 (19.7)	
Survey year, *n* (%)						0.053

^a^ Study cohort consisted of prostate cancer survivors having a Consumer Assessment of Healthcare Providers and Systems (CAHPS) survey ≥ 6 months from PCa diagnosis and first such survey was used; ^b^ SD: Standard deviation; ^c^ CAHPS: Consumer Assessment of Healthcare Providers and Systems; ^d^ PCa: Prostate cancer; *p*-values generated using Analysis of variance (ANOVA), Chi-square, and Fisher’s Exact test as appropriate; Cell sizes < 11 have been suppressed as per CMS policy of small cell sizes.

**Table 2:** Clinical characteristics of study cohort by race/ethnicity ^a^.

Variable	Non-Hispanic Whites (*n* = 5253)	Non-Hispanic Blacks (*n* = 851)	Hispanics (*n* = 595)	Non-Hispanic Asians (*n* = 386)	Other Races (*n* = 234)	*p*-Value
**Comorbidity count, *n* (%)**						<0.001
0	2348 (44.7)	304 (35.7)	242 (40.7)	165 (42.8)	95 (40.6)	
1	1812 (34.5)	318 (37.4)	224 (37.7)	139 (36.0)	81 (34.6)	
2	834 (15.9)	173 (20.3)	86 (14.5)	65 (16.8)	42 (18.0)	
3 or 4	259 (4.9)	56 (6.6)	43 (7.2)	17 (4.4)	16 (6.8)	
**Tumor grade, *n* (%)**						0.113
Well/moderately differentiated	2274 (43.3)	340 (40.0)	261 (43.9)	141 (36.5)	88 (37.6)	
Poorly differentiated	2794 (53.2)	479 (56.3)	310 (52.1)	233 (60.4)	>135 (>57.7)	
Undifferentiated/unknown	185 (3.5)	32 (3.8)	24 (4.0)	12 (3.1)	<11 (<4.7)	
**Lymph node involvement, *n* (%)**						<0.001
None	4863 (92.6)	782 (91.9)	>501 (>84.2)	>338 (>87.6)	>207 (>88.5)	
Regional lymph nodes/lymph nodes, NOS ^b^	60 (1.1)	12 (1.4)	<11 (<1.8)	<11 (<2.8)	<11 (<4.7)	
Unknown	330 (6.3)	57 (6.7)	83 (14.0)	37 (9.6)	16 (6.8)	
**Receipt of radiation as a part of initial treatment, *n* (%)**						0.003
No	3005 (57.2)	445 (52.3)	346 (58.2)	212 (54.9)	133 (56.8)	
Yes	2126 (40.5)	392 (46.1)	>238 (>40.0)	157 (40.7)	>90 (>38.5)	
Missing	122 (2.3)	14 (1.7)	<11 (<1.8)	17 (4.4)	<11 (<4.7)	
**Receipt of definitive surgery as a part of initial treatment, *n* (%)**						<0.001
No	3681 (70.1)	664 (78)	441 (74.1)	258 (66.8)	168 (71.8)	
Yes	1498 (28.5)	>176 (>20.7)	>143 (>24.0)	115 (29.8)	>55 (>23.5)	
Missing	74 (1.4)	<11 (<1.3)	<11 (1.8)	13 (3.4)	<11 (<4.7)	
**Risk of disease progression, *n* (%)**						<0.001
Low	933 (17.8)	154 (18.1)	98 (16.5)	63 (16.3)	39 (16.7)	
Intermediate	2838 (54)	488 (57.3)	293 (49.2)	206 (53.4)	129 (55.1)	
High	802 (15.3)	121 (14.2)	121 (20.3)	87 (22.5)	44 (18.8)	
Missing	680 (12.9)	88 (10.3)	83 (14)	30 (7.8)	22 (9.4)	
**Number of prior cancers other than prostate cancer, *n* (%)**						<0.001
0	4648 (88.5)	791 (93)	558 (93.8)	349 (90.4)	220 (94)	
≥1	605 (11.5)	60 (7.1)	37 (6.2)	37 (9.6)	14 (6.0)	

^a^ Study cohort consisted of prostate cancer survivors having a Consumer Assessment of Healthcare Providers and Systems (CAHPS) survey ≥ 6 months from PCa diagnosis and first such survey was used; ^b^ NOS: Not otherwise specified.

**Table 3:** Self-reported health status of study cohort by race/ethnicity ^a^.

Variable	Non-Hispanic Whites (*n* = 5253)	Non-Hispanic Blacks (*n* = 851)	Hispanics (*n* = 595)	Non-Hispanic Asians (*n* = 386)	Other Races (*n* = 234)	*p*-Value
**General health status, *n* (%)**						<0.001
Missing	137 (2.6)	36 (4.2)	20 (3.4)	<11 (<2.8)	<11 (<4.7)	
Excellent	427 (8.1)	34 (4.0)	43 (7.2)	>28 (>7.2)	13 (5.6)	
Very good	1562 (29.7)	184 (21.6)	112 (18.8)	79 (20.5)	>55 (>23.5)	
Good	1984 (37.8)	329 (38.7)	210 (35.3)	165 (42.8)	87 (37.2)	
Fair	906 (17.3)	228 (26.8)	177 (29.8)	88 (22.8)	52 (22.2)	
Poor	237 (4.5)	40 (4.7)	33 (5.6)	15 (3.9)	16 (6.8)	
**Mental health status, *n* (%)**						<0.001
Missing	140 (2.7)	30 (3.5)	22 (3.7)	14 (3.6)	0 (0)	
Excellent	1778 (33.9)	237 (27.9)	127 (21.3)	82 (21.2)	71 (30.3)	
Very good	1830 (34.8)	242 (28.4)	172 (28.9)	122 (31.6)	69 (29.5)	
Good	1147 (21.8)	214 (25.2)	175 (29.4)	120 (31.1)	63 (26.9)	
Fair	294 (5.6)	112 (13.2)	>88 (>14.8)	>37 (>9.6)	>20 (>8.5)	
Poor	64 (1.2)	16 (1.9)	<11 (<1.8)	<11 (<2.8)	<11 (<4.7)	

^a^ Study cohort consisted of prostate cancer survivors having a Consumer Assessment of Healthcare Providers and Systems (CAHPS) survey ≥ 6 months from PCa diagnosis and first such survey was used.

**Table 4:** Least-square mean estimates of patient experience measures by race/ethnicity for unadjusted, partially adjusted and fully adjusted models.

Outcome Variable	Non-Hispanic Whites LSE ± SSE			Non-Hispanic Blacks LSE ± SSE			Hispanics LSE ± SSE			Non-Hispanic Asians LSE ± SSE			Other Races LSE ± SSE		
	Unadjusted Model	Partially Adjusted Model	Fully Adjusted Model	Unadjusted Model	Partially Adjusted Model	Fully Adjusted Model	Unadjusted Model	Partially Adjusted Model	Fully Adjusted Model	Unadjusted Model	Partially Adjusted Model	Fully Adjusted Model	Unadjusted Model	Partially Adjusted Model	Fully Adjusted Model
Getting needed care (*n* = 5486)	88.46 ± 0.31	83.35 ± 1.15	80.54 ± 2.57	84.35 ± 0.84	81.32 ± 1.35	78.76 ± 2.64	83.02 ± 0.95	80.90 ± 1.40	78.38 ± 2.70	83.81 ± 1.17	80.97 ± 1.56	78.37 ± 2.78	84.87 ± 1.49	80.86 ± 1.82	78.05 ± 2.92
Getting care quickly (*n* = 6323)	73.14 ± 0.36	69.36 ± 1.33	66.47 ± 2.83	66.67 ± 0.89	64.83 ± 1.51	64.03 ± 2.91	67.93 ± 1.06	66.78 ± 1.61	62.77 ± 2.97	66.62 ± 1.32	64.64 ± 1.78	60.03 ± 3.08	70.7 ± 1.67	67.77 ± 2.06	64.39 ± 3.22
Doctor communication (*n* = 5487)	91.37 ± 0.25	88.55 ± 0.91	90.10 ± 2.02	91.94 ± 0.60	90.20 ± 1.04	92.06 ± 2.09	87.87 ± 0.73	86.82 ± 1.09	88.67 ± 2.11	88.91 ± 0.88	87.28 ± 1.20	88.94 ± 2.18	88.95 ± 1.15	86.50 ± 1.41	88.44 ± 2.29
Customer Service (*n* = 1968)	80.06 ± 0.72	75.93 ± 3.20	78.31 ± 5.42	79.71 ± 1.50	77.36 ± 3.42	79.71 ± 5.62	77.68 ± 1.69	75.88 ± 3.51	77.14 ± 5.63	72.62 ± 2.40	70.03 ± 3.83	70.71 ± 5.85	76.94 ± 2.99	74.31 ± 4.18	76.92 ± 6.08
Getting needed prescription drugs (*n* = 4131)	91.39 ± 0.38	87.29 ± 1.24	90.26 ± 3.09	86.43 ± 0.86	84.44 ± 1.41	88.55 ± 3.19	86.47 ± 0.95	84.75 ± 1.47	87.33 ± 3.16	87.28 ± 1.22	84.63 ± 1.64	87.18 ± 3.28	87.20 ± 1.67	84.62 ± 2.00	87.39 ± 3.44

LSM: Least Square Mean; SE: Standard Error; Unadjusted models: Not adjusted for any covariate; Partially adjusted models: Adjusted for case-mix variables (age when responded to survey, proxy answering questions for respondent, mental health status, general health status, low-income subsidy, dual eligibility, and education). Fully adjusted models: Adjusted for case-mix variables, adjusted for plan type, prescription drug plan, marital status, geographic region of residence at the time of CAHPS survey, urban/rural residence status, Census Tract Poverty Indicator for neighborhoods, survey year, current smoking status, tumor grade, lymph node involvement, risk of disease progression, receipt of radiation as a part of initial treatment, receipt of definitive surgery as a part of initial treatment, number of prior cancers other than prostate cancer, time between prostate cancer diagnosis and CAHPS survey, comorbidity count.

## Data Availability

SEER-CAHPS data are available from the National Cancer Institute upon request and signing data use agreement.

## Supplementary Materials

The following supporting information can be downloaded at: https://www.mdpi.com/article/10.3390/curroncol29110659/s1, Table S1 provides variance inflation factor for each variable included in the models examining associations of race/ethnicity with each patient care experience measure. Table S2 provides a comparison of the results of the study by Halpern et al., 2018 [14] to the CMS Office of Minority Health Report assessing trends in racial inequities in healthcare from 2009–2018 among a nationally representative sample of MA enrollees. e-Methods provides details about outcome variable, covariates and statistical analyses [52,53,54,55,56].
