# Supplementary material for: Racial/Ethnic Disparities in Patient Care Experiences among Prostate Cancer Survivors: A SEER-CAHPS Study
Source: Curr Oncol. 2022 Nov 1;29(11):8357–73. doi: 10.3390/curroncol29110659 (PMC9689524; doi:10.3390/curroncol29110659)
Supplement: Supplementary file 1 [file curroncol-29-00659-s001.zip › curroncol-1936053-supplementary.pdf]

Table S1. Variance inflation factor for each variable included in the models examining associations of race/ethnicity with each patient care experience measure.

Variable	Getting needed care	Getting care quickly	Doctor communication	Customer service	Getting needed prescription drugs
Age when responded to survey	1.18	1.19	1.19	1.20	1.20
Time between PCa[a] diagnosis and CAHPS[b] survey	1.48	1.48	1.49	1.47	1.50
Health plan type at the time of CAHPS[b] survey	2.86	2.88	2.92	2.43	1.09
Prescription drug plan	2.97	3.00	3.02	2.42	.
Education level	1.30	1.35	1.31	1.32	1.28
Proxy answered questions for the respondent	1.21	1.25	1.22	1.23	1.26
Low-income subsidy	2.39	2.45	2.42	3.31	2.34
Dual eligibility for Medicare and Medicaid	2.32	2.36	2.33	3.22	2.20
Marital Status	1.09	1.08	1.09	1.11	1.10
Geographic region when answering to CAHPS[b] survey	1.09	1.09	1.10	1.09	1.09
Census tract poverty indicator	1.20	1.20	1.20	1.19	1.20
Rurality	1.08	1.07	1.07	1.06	1.06
Smoking status	1.08	1.15	1.13	1.10	1.07
Survey year	1.64	1.64	1.66	1.54	1.67
Comorbidity count	1.19	1.19	1.20	1.20	1.17
Tumor grade	1.04	1.04	1.04	1.07	1.05
Lymph node involvement	1.11	1.10	1.11	1.11	1.10
Receipt of radiation as a part of initial treatment	1.14	1.13	1.12	1.17	1.13

Variable	Getting needed care	Getting care quickly	Doctor communication	Customer service	Getting needed prescription drugs
**Receipt of definitive surgery as a part of initial treatment**	1.26	1.25	1.24	1.28	1.22
**Risk of disease progression**	1.09	1.08	1.08	1.10	1.09
**Number of prior cancers other than prostate cancer**	1.02	1.02	1.02	1.02	1.02
**General health status**	1.51	1.55	1.52	1.55	1.50
**Mental health status**	1.41	1.45	1.43	1.49	1.44
**Race/ethnicity**	1.13	1.13	1.12	1.14	1.16

[a]PCa: Prostate cancer; [b]CAHPS: Consumer Assessment of Healthcare Providers and Systems; [c]N/A: Not applicable

Table S2. Comparison of the results of the study by Halpern et al., 2018 to the CMS Office of Minority Health Report assessing trends in racial inequities in healthcare from 2009-2018 among a nationally representative sample of MA enrollees.

	Non-Hispanic Blacks			Hispanics			Non-Hispanic Asians			Other		
**PCEs common in both the studies compared**	**Halpern et al., 2018***	**CMS report CY 2009****	**CMS report CY 2018****	**Halpern et al., 2018***	**CMS report CY 2009****	**CMS report CY 2018****	**Halpern et al., 2018***	**CMS report CY 2009****	**CMS report CY 2018****	**Halpern et al., 2018***	**CMS report CY 2009****	**CMS report CY 2018****
**Getting needed care**	+	-	0	0	-	-	-	-	-	0	0	0
**Getting care quickly**	0	-	-	-	-	-	-	-	-	0	0	0
**Customer service**	+	-	0	0	-	0	0	-	-	0	0	0

* Halpern MT, Urato MP, Lines LM, et al. Healthcare experience among older cancer survivors: Analysis of the SEER-CAHPS dataset. *J Geriatr Oncol* 2018; 9: 194–203.

**Martino S, Elliott M, Haas A, et al. *Trends in Racial, Ethnic, Sex, and Rural-Urban Inequities in Health Care in Medicare Advantage: 2009-2018*, www.rand.org/health-care, (2021, accessed 4 April 2022).

+: Better than non-Hispanic Whites

-: Worse than non-Hispanic Whites

0: Similar to non-Hispanic Whites

e-Methods

This file provides details about deriving scores for composite measures, handling missingness, additional details regarding covariates, and statistical analyses. It also provides results for comparison between a previous study evaluating racial/ethnic disparities in patient care experiences of prostate cancer survivors and our study findings and CMS report on disparity trends.

Outcome variable

Composite measure scores in CAHPS are derived from responses to individual questions contributing to each composite measure [1]. For example, the composite measure of 'getting needed care' is derived from responses to a set of two CAHPS questions 'How often was it easy to get appointments with specialists?' and 'How often was it easy to get the care, tests, or treatment you thought you needed through Medicare?'. Likewise, composite measure scores for 'doctor communication' is derived from responses to a set of four CAHPS questions, while composite measure scores for 'getting care quickly', 'customer service', and 'getting needed prescription drugs' are each derived from responses to separate sets of three CAHPS questions each. A person would have a valid response for a given PCE composite measure if the person is eligible to answer questions contributing to that PCE composite measure and has answered at least one of these questions.

Depending on their health plan or specific situations, enrollees may be ineligible to answer certain survey questions and hence have a missing score for a PCE. If a person is ineligible to answer all the questions contributing to a PCE composite measure, that person would have a missing score for that PCE composite measure. For example, those not enrolled in a prescription drug plan would be ineligible to answer all CAHPS questions related to prescription drugs and would have a missing composite score of 'getting needed prescription drugs'. Similarly, those enrollees who never sought

information/help from health plan's customer service would have a missing composite score for 'customer service' or those who did not visit personal doctor would have a missing score for 'doctor communication'. Among individuals who were eligible answer questions for a given PCE composite measure, the proportion of missing values ranged from 2% for 'getting care quickly' and up to 15% for 'customer service'. Final analysis only included non-missing responses on PCE composite measures.

Some studies have found greater use of extremes of the scales with respect to the 0–10 global ratings among African Americans and Latinos [2,3], providing evidence against using central tendency measures of these ratings (e.g. mean) to assess racial/ethnic disparities. By CMS's recommendation, we did not study racial/ethnic disparities in any of global ratings as outcome.

Covariates

Risk of disease progression was based on prostate specific antigen (PSA) level, Gleason score, and tumor stage, categorized as low, intermediate, and high as per National Comprehensive Cancer Network (NCCN) guidelines [4]. Definitive surgery was defined as radical prostatectomy, prostatectomy with resection in continuity with other organs, or pelvic exenteration. CAHPS asks responders about 4 types of comorbidities (heart conditions, stroke, chronic obstructive pulmonary disease, and diabetes); thus, the maximum comorbidity count is 4. We used RUCC derived from county FIPS codes of survivors' residence to determine rural-urban status [5]. Counties with RUCC of 0-3 were considered urban while areas having RUCC from 4-9 were categorized as rural. Geographic regions were defined as per the United States Census Bureau guidance [6].

Statistical analyses

We conducted multicollinearity testing for the covariates included in the models. A Variance Inflation Factor (VIF) of >10 for any covariate was considered as a sign of multicollinearity. No significant multicollinearity problems were found (Table S1).

Comparison to previous study in PCa survivors using SEER-CAHPS

Using 2007-2015 SEER-CAHPS data, this study builds on the existing evidence [7] of racial/ethnic disparities in PCEs among PCa survivors. It is important to note that the previous study dichotomized the PCE outcomes as 'high/not high', while our study analyzed PCE scores as continuous measures, which has greater statistical power [8]. Moreover, survey questions for many of the PCEs have evolved over the years, from 2000 to 2015 [9], making it impossible to directly compare with previous study findings. Nonetheless, the direction of many of the adjusted associations observed in our study were consistent with those previously reported [7]. For instance, like previous study [7], we found that compared to NHW PCa survivors, Hispanic and NHA PCa survivors had poorer experiences with getting care quickly and getting needed Rx. However, there were some inconsistencies as well, especially regarding PCEs in NHBs.

The previous study reported that compared to NHW PCa survivors, Hispanic and NHA PCa survivors had poorer experiences with doctor communication and NHA PCa survivors had poorer experiences with getting needed care, but we did not observe these associations. Moreover, none of the adjusted associations found by the previous study among NHB PCa survivors were observed in our study and vice-versa. While the previous study reported better experiences for getting needed care and customer service by NHB compared to NHW PCa survivors, we did not observe any of these associations. Instead, we found that compared to NHW, NHB PCa survivors had better experiences with doctor communication and worse experience with getting care quickly. Our study

revealed additional disparities not found in the previous study. For instance, NHA compared to NHW PCa survivors had poorer experiences for customer service which have not been reported by the previous study [7]. The differences between the two studies may be due to changes in racial/ethnic disparities in care experiences over time but may also be a result of changes in the CAHPS survey questions.

Comparing findings from Halpern et al. with the CMS Office of Minority Health Report for trends in racial inequities in healthcare from 2009-2018 among a nationally representative sample of MA enrollees

CMS Office of Minority Health Reports assessed trends in racial inequities in healthcare from 2009-2018 among a nationally representative sample of MA enrollees [10].Three PCE composite measures (e.g. getting needed care, getting care quickly, customer service) were reported in both the previous study and CMS reports. Comparing the results of the previous study to the reports shows improved scores in NHBs, worsening scores in NHAs, mixed results in Hispanics, but no changes in other races group (Table S2). However, these reports also used linear mean scoring in assessing racial disparities as ours, and therefore not directly comparable to the previous PCa study [7]. Moreover, data on PCa survivors were not separately reported. Thus, it is unclear whether this reflects a change in the reporting trend or differences between PCa survivors and the general MA population. Further research using data from other sources is needed to confirm if patient experience has changed among PCa survivors in more recent years.

Reference

1. Chawla, N.; Urato, M.; Ambs, A.; Schussler, N.; Hays, R.D.; Clauser, S.B.; Zaslavsky, A.M.; Walsh, K.; Schwartz, M.; Halpern, M.; et al. Unveiling SEER-CAHPS®: A New Data Resource for Quality of Care Research. *J. Gen. Intern. Med.* 2015, *30*, 641–650, https://doi.org/10.1007/s11606-014-3162-9.

2. Weinick, R.M.; Elliott, M.N.; Volandes, A.E.; Lopez, L.; Burkhart, Q.; Schlesinger, M. Using Standardized Encounters to Understand Reported Racial/Ethnic Disparities in Patient Experiences with Care. *Heal. Serv. Res.* **2010**, *46*, 491–509, https://doi.org/10.1111/j.1475-6773.2010.01214.x.
3. Martino, S.C.; Weinick, R.M.; Kanouse, D.E.; Brown, J.A.; Haviland, A.M.; Goldstein, E.; Adams, J.L.; Hambarsoomian, K.; Klein, D.J.; Elliott, M.N. Reporting CAHPS and HEDIS Data by Race/Ethnicity for Medicare Beneficiaries. *Heal. Serv. Res.* **2012**, *48*, 417–434, https://doi.org/10.1111/j.1475-6773.2012.01452.x.
4. National Comprehensive Cancer Network (NCCN). Prostate Cancer Early Stage. 2020. Available online: https://www.nccn.org/patients/guidelines/content/PDF/prostate-early-patient.pdf (accessed on 15 September 2021).
5. Parker, T. Measuring Rurality: Rural-Urban Continuum Codes; US Department of Agriculture: Washington, DC, USA, 2012. Available online: http://www.ers.usda.gov/data-products/rural-urban-continuum-codes.aspx (accessed on 10 August 2012).
6. United States Census Bureau. Geographic Levels. Available online: https://www.census.gov/programs-surveys/economic-census/guidance-geographies/levels.html (accessed on 22 April 2022).
7. Halpern, M.T.; Urato, M.P.; Lines, L.M.; Cohen, J.B.; Arora, N.K.; Kent, E.E. Healthcare experience among older cancer survivors: Analysis of the SEER-CAHPS dataset. *J. Geriatr. Oncol.* **2018**, *9*, 194–203, doi:10.1016/j.jgo.2017.11.005.
8. National Cancer Institute. SEER-CAHPS Analytic Guidance. 2020. Available online: https://healthcaredelivery.cancer.gov/seer-cahps/researchers/approaches_guidance.html (accessed on 15 February 2020).
9. National Cancer Institute. CAHPS Data Documentation. Available online: https://healthcaredelivery.cancer.gov/seer-cahps/aboutdata/documentation.html (accessed 24 March 2022).
10. Martino S, Elliott M, Haas A, Klein D, Hambarsoomian K, Haviland A, et al. Trends in Racial, Ethnic, Sex, and Rural-Urban Inequities in Health Care in Medicare Advantage: 2009-2018. 2021. Available online: www.rand.org/health-care (accessed 4 April 2022).
